# Our Advertising Department

**Published:** 1878-11-20

**Authors:** 


### OUR ADVERTISING DEPARTMENT.

Our Advertising Department has been gradually enlarging. We mentioned in a former occasion, in response to a complaint on this point that our reading matter was not encroached upon by the advertisements.

